# Exploring the Knowledge Regarding Mucormycosis and Its Management Among a Cohort of Dental Undergraduates in India: A Cross-Sectional Survey

**DOI:** 10.7759/cureus.38918

**Published:** 2023-05-12

**Authors:** Priya Deepa Lakshmi K, Saravanan N, Bharath C, Santhakumari S, Prabavathi M, Aditi Chattaraj

**Affiliations:** 1 Public Health Dentistry, Vinayaka Mission's Sankarachariyar Dental College, Vinayaka Mission’s Research Foundation (Deemed to be University), Salem, Tamil Nadu, IND; 2 Public Health Dentistry, Vinayaka Mission’s Sankarachariyar Dental College, Vinayaka Mission’s Research Foundation (Deemed to be University), Salem, Tamil Nadu, IND; 3 Public Health Dentistry, Vinayaka Mission's Sankarachariyar Dental College, Vinayaka Mission's Research Foundation (Deemed to be University), Salem, Tamil Nadu, IND; 4 Dentistry, Vinayaka Mission’s Sankarachariyar Dental College, Vinayaka Mission’s Research Foundation (Deemed to be University), Salem, Tamil Nadu, IND

**Keywords:** survey, mucormycosis, management, knowledge, dental undergraduates

## Abstract

Introduction: Mucormycosis is an angioinvasive fungal infection associated with a high mortality rate in both low- and middle-income countries. A dentist plays a crucial role and first line in the diagnosis and treatment of mucormycosis since the majority of the site of infection is the rhino cerebral or rhino maxillary area. The present study was designed to ascertain knowledge about mucormycosis and its management among a sample of dental undergraduates in India.

Materials and methods: A self-administered questionnaire covering demographic details, knowledge relating to underlying disease and risk factors (10 items), clinical features and diagnosis (8 items), and management of mucormycosis (six items) was employed. Responses were recorded on a dichotomous scale. Data analysis was done using SPSS 20 (SPSS Inc., Chicago, IL, USA). The mean and standard deviation for correct answers and knowledge levels were determined.

Results: A total of 437 respondents were included. Classification of participants based on the level of correct knowledge demonstrated that the majority of students had good knowledge (232, 53.1%). Comparison of the same based on the college type showed significant differences for only clinical features, diagnosis (p=0.002), and management (p=0.035) whereas no significance was seen for gender. Correlation by Karl’s Pearson correlation coefficient revealed a significant positive correlation between the entire knowledge scores.

Conclusion: The study portrays adequate knowledge among dental interns that can be used to modify preventive care measures to lessen the public health emergency. Stakeholders can take the necessary action to spread knowledge about mucormycosis through training workshops and continuing dental education programs to combat the health crisis.

## Introduction

Humans learn the pleasure of health from the bitterness of disease. As we set foot in the 21st century, mankind is being endangered by multiple ailments which have forced humanity to put the world on hiatus. Apart from COVID-19, one such threat that rose is mucormycosis, an angioinvasive infection acquired by a saprophytic fungus that has proved to be ubiquitous [1-2].

The annual incidence rate of mucormycosis on a global scale ranges from 0.005 to 1.7 per 100 million population [3]. Recently, it is associated with a high mortality rate in both low- and middle-income countries [2]. Around 140 cases per million inhabitants are affected by this incursive infection in India almost 80 times higher than in developed countries [4]. As of May to July 2021 India has confirmed more than 47,000 new cases of mucormycosis compared with the rest of the nations [5]. The sudden surge of the disease is due to the increasing prevalence of comorbid people, iron overload, and overdosage with injudicious use of corticosteroids among COVID-19 patients [1, 6].

A literature search revealed that a prospective multicenter study done by Patel et al. [7] identified that the knowledge deficit among medicos is the problem encountered in the identification and management of mucormycosis. Therefore, improvization in diagnostic methods, stewardship of antifungal therapy, and awareness of medical staff are mandatory. Dental professionals play the part of team members and have a salient role in treating this mycotic condition both at a primary and tertiary level of care. Since they might encounter patients of this condition in their out-patient diagnosis concerning the maxillofacial region and some cases reported post-tooth extraction [8]. These cases were found to be approaching dentists more comparatively than general physicians because of the area of involvement. Few studies have evaluated the cognizance level of mucormycosis among medical professionals [9], nursing students [10], and the general population [11], not one research so far has interrogated the same among dental undergraduates, especially interns. For these reasons, the present study was designed to ascertain knowledge about mucormycosis and its management among a sample of both government and private dental college undergraduates in India.

## Materials and methods

Study design and population

Using a convenient snowball sampling technique, this cross-sectional questionnaire-based study was conducted from October to December 2021 among dental undergraduates in India who are pursuing their internship in the academic year 2020-2021.

Ethical approval

Ethical clearance for this study was obtained from the Ethical Committee of the Institutional Review Board, Vinayaka Mission’s Sankarachariyar Dental College, Salem (VMSDC/IEC/Approval No.213).

Data collection procedure

Data were gathered via a self-administered online survey (through Google form). When study participants clicked on the link (shared via WhatsApp), they were taken to an information sheet describing the study's goal and purpose. The respondents who had consented to participate and were willing to do so were moved on to the next section of the survey.

Sample size calculation

Before the data collection, sample size estimation was made using the formula for the infinite population, and it was found to be 384 by accomplishing a confidence level of 95%, with a margin error of 5%, and taking a 50% response distribution into consideration. Subsequently, in order to calculate the finite sample size by considering the permitted seats of students by the Dental Council of India during 2016-2017 [12], is around 26,440 whereas the admitted seats are about 18,687. Therefore the sample is estimated by using the formula:

 N = Sample Size (SS)/ 1+ {(SS-1)/ Population} 

 N = 384 /1 + {383 /18,687} =377.

Data collection tool

The questionnaire was developed based on World Health Organization (WHO) [3] and Center for Disease and Control guidelines [13]. The questionnaire encompassed four sections: demographic details regarding age, gender, and type of college (Government or Private) were gathered in the first section. The next three sections consisted of questions on knowledge relating to underlying disease and risk factors (Q1-10, 10 items), clinical features and diagnosis (Q11-18, 8 items), and management of mucormycosis (Q19-24, 6 items) respectively [7, 9]. The responses were recorded on a dichotomous scale (Yes/No). For each item, scoring was done as 1 for the correct answer and 0 for the incorrect answer. The total scores were obtained by summing the correct answer scores (cut-off score=17) and a higher cut-off score indicated a higher level of knowledge [9].

Pretesting of questionnaire

Five academic proficient experts in this area reviewed and validated the preliminary draft of the study questionnaire for its content, design, materiality, clarity, and understandability. Then the final version was constructed based on the experts’ opinions followed by pilot testing carried out on 30 students to check the reliability of the questionnaire (Cronbach’s alpha coefficient = 0.74).

Statistical analysis

Data analysis was done using IBM Statistical Package for Social Sciences version 20.0 (SPSS Inc., Chicago, IL, USA). By using the mean and standard deviation approach, cataloguing the knowledge levels was done by low and high. A comparison of the mean correct knowledge scores and levels of knowledge was done based on the type of college by using an independent sample t-test. Pearson’s Coefficient was used for evaluating the correlations among knowledge scores. For all statistical analyses, p ≤ 0.05 was considered significant.

## Results

A total of 437 respondents were included from the Northern, Southern, Western, and Eastern regions of India. Overall, 56 dental colleges located in 17 different states were represented in the study. Out of 437 study population, 227 (51.9%) belonged to Government and the remaining 210 (48.1%) were from private colleges. State wise, the study participants are distributed as follows: New Delhi-34, Madhya Pradesh-48, Uttar Pradesh-32, Haryana-04, Bihar-09, Rajasthan-10, Tamil Nadu-77, Karnataka-43, Kerala-18, Andhra Pradesh-10, Telangana-04, Maharastra-57, Gujarat-19, Goa-10, West Bengal-30, Odisha-28, and Jharkhand-04. Nearly three fourth of the respondents (N=328, 75.1%) were females. The respondents’ ages ranged between 22 and 29 years [mean ± standard deviation (SD) =23 ± 1.36]. Most of the participants 94.3% (N=412) belonged to the age group 22-25 years.

Correct answers to the questions and assessment of item-wise mean scores based on gender and college type are revealed in Table 1.

**Table 1:** Total number of correct responses [N (%)] and comparison of item wise mean score stratified based on gender and college type. *Statistically significant (p < 0.05)

Items	Correct answer N (%)	Gender Mean±SD		p-value	College type Mean±SD		p-value
		Male	Female		Government	Private	
Q1 Mucormycosis is a contagious disease	No 263(60.2)	0.61±0.489	0.60±0.491	0.752	0.63±0.484	0.57±0.496	0.213
Q2 Mucormycosis is a gender-oriented condition	No 393(89.9)	0.85±0.356	0.91±0.280	0.065	0.91±0.284	0.89±0.319	0.365
Q3 Mucormycosis can be contracted by people of any age group	Yes 352(80.5)	0.75±0.434	0.82±0.382	0.106	0.78±0.418	0.84±0.369	0.098
Q4 Risk group includes people with neutropenia	Yes 326(74.6)	0.66±0.476	0.77±0.419	0.018*	0.74±0.440	0.75±0.433	0.769
Q5 Risk group includes people with organ transplant	Yes 334(76.4)	0.81±0.396	0.75±0.434	0.223	0.76±0.429	0.77±0.421	0.736
Q6 Risk group includes people with epilepsy	No 318(72.8)	0.70±0.462	0.74±0.441	0.411	0.77±0.421	0.68±0.467	0.035*
Q7 Risk group includes people with diabetic ketoacidosis	Yes 394(90.2)	0.94±0.229	0.89±0.317	0.080	0.91±0.284	0.89±0.313	0.454
Q8 Causative organism is found more in the air than soil.	No 190(43.5)	0.47±0.501	0.42±0.495	0.422	0.45±0.499	0.42±0.495	0.524
Q9 Inhalation of fungal spores is the only way of acquiring mucormycosis	No 198(45.3)	0.55±0.500	0.42±0.494	0.018*	0.46±0.500	0.44±0.498	0.680
Q10 Covid-19 patients on steroid therapy are more prone to mucor infection	Yes 421(96.3)	0.93±0.262	0.98±0.154	0.018*	0.96±0.185	0.96±0.192	0.874
Q11 Pterygopalatine fossa is the largest reservoir and channel for the spread in mucormycosis	Yes 367(84.0)	0.74±0.39	0.87±0.335	0.001*	0.78±0.418	0.91±0.288	0.000*
Q12 In head and neck, common reported site of mucormycosis is sinuses	Yes 395(90.4)	0.88±0.326	0.91±0.284	0.345	0.89±0.314	0.92±0.273	0.302
Q13 Major clinical feature of mucormycosis is necrosis	Yes 399(91.3)	0.94±0.246	0.91±0.293	0.332	0.93±0.264	0.90±0.301	0.353
Q14 Mucormycosis is classified based on the organ affected	Yes 309(70.7)	0.67±0.472	0.72±0.450	0.324	0.67±0.471	0.75±0.435	0.074
Q15 Direct microscopy of potassium hydroxide wet mounts is used for a rapid presumptive diagnosis of mucormycosis	Yes 350(80.1)	0.70±0.462	0.84±0.371	0.002*	0.79±0.406	0.81±0.394	0.666
Q16 Serological test is used for the diagnosis of mucormycosis	No 110(25.2)	0.28±0.449	0.24±0.430	0.515	0.28±0.449	0.22±0.418	0.197
Q17 Polymerase chain reaction techniques can help in identifying the fungal species in culture specimens	No 114(26.1)	0.22±0.416	0.27±0.447	0.265	0.27±0.444	0.25±0.435	0.698
Q18 Magnetic resonance imaging is better suited than computerized tomography in differentiating rhino-orbital-cerebral mucormycosis from other disease with similar clinical presentation	Yes 376(86.0)	0.87±0.336	0.86±0.351	0.699	0.80±0.400	0.92±0.266	0.000*
Q19 Mucormycosis is always curable in Covid-19 patients.	No 233(53.3)	0.50±0.502	0.55±0.499	0.363	0.52±0.501	0.54±0.499	0.697
Q20 Primary antifungal therapy used for mucormycosis is liposomal amphotericin B	Yes 419(95.9)	0.94±0.229	0.96±0.188	0.402	0.95±0.215	0.97±0.180	0.428
Q21 Voriconazole drug can be used in treatment of mucormycosis	No 102(23.3)	0.27±0.444	0.22±0.417	0.353	0.21±0.406	0.26±0.441	0.176
Q22 There is no standard duration of treatment for mucormycosis	Yes 353(80.8)	0.82±0.389	0.80±0.397	0.790	0.88±0.330	0.73±0.443	0.000*
Q23 Surgical debridement is the only option to prevent the recurrence of mucormycosis	No 126(28.8)	0.26±0.439	0.30±0.458	0.404	0.35±0.479	0.22±0.415	0.002*
Q24 Reversal of immunosuppression along with surgery and appropriate early antifungal agents is an important pillar of therapy for mucormycosis	Yes 412(94.3)	0.93±0.262	0.95±0.222	0.402	0.93±0.257	0.96±0.203	0.215

In 24-item questions, the mean number of correct answers was 17.35 (SD = 2.23) for all the participants. Among all the questions, the least proportion of students answering correctly of the items were Q16 (N=110, 25.2%) and Q21 (N=102, 23.3%). Based on gender with correct knowledge revealed that females had better knowledge as compared to males, a significant difference was noted only for questions 4,9,10,11, and 15 (p<0.05). On the other hand, when college type was considered, the majority of the questions had the highest mean score for government college students compared to a private college. However, significance was observed for questions 6, 11, 18, 22, and 23 (p<0.05). 

On the other hand, when correct mean scores for knowledge were compared based on type of college, the correct mean scores for knowledge about clinical features and diagnosis (p=0.014) and management of mucormycosis (p=0.05) only illustrated a significant difference. Likewise, only clinical features and diagnosis showed a significant difference for gender (p=0.015) (Table 2).

**Table 2:** Mean±SD of the correct answer for total knowledge, underlying disease, risk factors, clinical features, diagnosis, and management based on the type of college. SD, standard deviation; CI, confidence interval *Statistically significant (p < 0.05)

Variables		Total knowledge Mean±SD	Underlying disease and risk factors Mean±SD	Clinical features and diagnosis Mean±SD	Management Mean±SD
Gender	Male	16.28±2.445	7.28±1.592	5.29±1.363	3.71±0.906
	Female	16.71±2.174	7.30±1.412	5.62±1.143	3.78±0.938
	P-value	0.082	0.854	0.015*	0.494
	Mean difference	-0.432	-0.030	-0.325	-0.070
	95% CI (lower-upper)	-0.920-0.056	-0.347-0.287	-0.586—0.064	-0.272-0.132
College type	Government	16.62±2.269	7.37±1.378	5.40±1.280	3.84±0.922
	Private	16.58±2.234	7.21±1.536	5.69 ±1.109	3.67±0.931
	p-value	0.868	0.251	0.014*	0.050*
	Mean difference	0.036	0.160	-0.285	0.172
	95% CI (lower-upper)	-0.346 -0.460	-0.114-0.434	-0.511—0.059	-0.003-0.388

Classification of study participants based on the level of correct knowledge demonstrated the majority of students had good and adequate knowledge (232, 53.1%). Comparison of the same based on the college type showed significant differences for only clinical features, diagnosis (p=0.002), and management (p=0.035) whereas no significance was seen based on gender (Table 3).

**Table 3:** Association of the levels of knowledge based on college type. *Statistically significant (p < 0.05)

Factors	Levels of total knowledge N (%)		p-value	Underlying disease and risk factors N (%)		p-value	Clinical features and diagnosis N (%)		p-value	Management N (%)		p-value	
	Poor	Good		Poor	Good		Poor	Good		Poor	Good		
Gender													
Male	55 (50.4)	54 (49.6)	0.228	33 (30.3)	76 (69.7)	0.370	21 (19.3)	88 (80.7)	0.222	48 (44.0)	61 (56.0)	0.411	
Female	150 (45.7)	178 (54.3)		92 (28.0)	236 (72.0)		51 (15.5)	277 (84.5)		138 (42.1)	190 (57.9)		
College type	105 (46.3)	122 (53.7)	0.425	64 (28.2)	163 (71.8)	0.463	49 (21.6)	178 (78.4)	0.002*	87 (38.3)	140 (61.7)	0.035*	
Government													
Private	100 (47.6)	110 (52.4)		61 (29.0)	149 (71.0)		23 (11.0)	187 (89.0)		100 (47.6)	110 (52.4)		

Correlation by Karl’s Pearson correlation coefficient revealed a significant positive correlation between the entire knowledge scores, i.e. total, underlying disease, risk factors, clinical features, diagnosis, and management knowledge (Table 4).

**Table 4:** Correlation coefficient between underlying disease, risk factors, clinical features, diagnosis, management, and total knowledge scores of mucormycosis. *Statistically significant (p < 0.05)

	Underlying disease and risk factors knowledge scores	Clinical features and diagnosis knowledge scores	Management knowledge scores	Total knowledge scores
Underlying disease and risk factors knowledge scores	1	0.038	0.153*	0.735*
Clinical features and diagnosis knowledge scores	0.038	1	0.004	0.568*
Management knowledge scores	0.153*	0.004	1	0.520*
Total knowledge scores	0.735*	0.568*	0.520*	1

## Discussion

The current study provides an outline of mucormycosis erudite among young professionals of dentistry across India. The extent of knowledge and awareness has been indicated as a rudimentary step in growing an optimistic approach toward the disease, its sequelae, and prevention.

In our study, we included 437 dental interns which were slightly higher than the study by Hasan et al. [14] on Bangladesh healthcare workers (N=422). A convenient snowball sampling technique was used due to the strategic ramifications, which may also be the reason why only a few samples are displayed. In recent times [15], a growing trend of women among dental professionals creates a domineering that females be considered as dynamic sophisticated potential for the future. This has been recognized in the current study as well, where the majority of the study participants were females (N=328, 75.1%).

The overall knowledge of mucormycosis in this group was rather adequate. This finding was not consistent with the study done by Oldaele et al. [9] among Nigerian resident doctors. This could be attributed to the students' urge in learning about mucor and also increased training in the diagnosis when it comes to oral manifestations of the condition associated with COVID-19.

When the knowledge of mucormycosis was analyzed, it was noted that the majority of students know that “COVID-19 patients on steroid therapy are more prone to mucor infection” suggesting of steroid treatment was a risk factor during the treatment of COVID-19 patients. These findings do concur with a multicentric study conducted by Jayagayathri et al. [11] among patients attending tertiary care hospitals in South India. However, in one previous systematic review and meta-analysis of case reports by Jeong et al. [16] it has been described that corticosteroid use did not appear to be an independent risk factor.

In our study, we made a step ahead by comparing variables like gender and type of college with correct responses. When gender comparison was done for knowledge scores, a higher mean score was observed among females (16.71 ± 2.17) as compared to males (16.28 ± 2.45). Likewise, when levels of correct knowledge were taken into account more females (54.3%) had good knowledge. This is consistent with the study by Nebhinani and Saini [17] which reported that female healthcare workers were perceptually sound compared with their counterparts.

Surprisingly, only 23.3% of the students had responded correctly that the Voriconazole prophylaxis cannot be used in treatment as it results in an increased frequency of mucormycosis cases after treatment. This shows the lacunae in knowledge among dental professionals regarding the use of improper antifungals for management.

When college type was taken into consideration, the mean total knowledge score (16.62 ± 2.269), underlying disease and risk factors (7.37 ± 1.378), and management (3.84 ± 0.922) were slightly higher among Government college interns which could be because of an increase reporting mucor cases. This finding was in keeping with a study conducted by Kabir et al. [18] described that public health workers are more proficient in disease insight. Moreover to our knowledge, no study has evaluated knowledge concerning underlying disease, risk factors, clinical features, diagnosis, and management of mucor infection among dental interns at various dental institutions across India.

Strength and limitations

This study has the following incontrovertible drawbacks the use of convenience sampling methods that may prevent the generalization of results. The cross-sectional design of the study can hinder the establishment of causal relationships and emphasizes the need for prospective longitudinal studies. The questionnaire used in this survey has been pre-tested, but this may limit the comparison of results with other surveys. In addition, the data was collected through self-reports which could cause bias. Also, since this study is not aimed at students across the country, we recommend that you do more research with more samples at different institutions in India. 

## Conclusions

The findings of this study prospect adequate knowledge among dental interns that can be used to modify the preventive measures to lessen the public health emergency. The stakeholders can therefore take the necessary action to spread knowledge about mucormycosis through deliberated teaching strategies, workshops, and continuing dental education programs in order to combat the health crisis. Thus, there is an imperative need for healthcare providers to become more aware of this, and specific efforts must be made to raise awareness among the general public.
